# Radiological and clinical aspect of Caudal regression syndrome associated with dorsal hemivertebra without maternal diabetes

**DOI:** 10.1016/j.radcr.2024.05.002

**Published:** 2024-06-06

**Authors:** Hassan Kadri, Mazen Dughly, Mohamad Shehadeh Agha, Raed Abouharb, Rostom Mackieh, Sameer Bakleh, Thea Kadri

**Affiliations:** a Department of Neurosurgery, Children's University Hospital, Faculty of Medicine, Damascus University; b Department of Neurology and Pediatrics, Children's University Hospital, Faculty of Medicine, Damascus University; c Department of Neuroradiology DNH, Damascus, Syria; d Department of Biology, George Washington university, Washington DC, USA

**Keywords:** Caudal regression syndrome (CRS), Hemivertebra, Scoliosis, Sacral agenesis

## Abstract

Caudal regression syndrome (CRS) is a rare genetic disorder affecting less than 0.1%-0.5% of newborns that manifests as the total or partial absence of lower vertebral structures including the sacral spine. The etiology of CRS remains elusive, but there is compelling evidence supporting a genetic predisposition and a correlation with maternal diabetes. This study presents the case of a 7-year-old girl exhibiting symptoms consistent with CRS including lower limb deficits, abnormal gait, urinary incontinence, and scoliosis. The findings from an MRI scan revealed notable anomalies such as hemivertebra in the dorsal spine, renal deformities, and the absence of secondary neurulation elements in the spine. We chose to delay the hemivertebra surgery because the scoliosis was not highly pronounced. Rather, we directed the child to the urology department for the management of her kidney deformities. This case contributes to the understanding of CRS and underscores the importance of comprehensive diagnostic approaches in elucidating its complex manifestations.

## Introduction

Caudal regression syndrome (CRS) is a rare genetic disorder affecting less than 0.1%-0.5% of newborns and characterized by the total or partial absence of lower vertebral structures including the sacral and thoracolumbar spine. It falls under spinal dysraphism—a disorder resulting from disruptions in spinal cord development with potential causes including genetic predisposition, maternal diabetes, and vascular hypoperfusion [1], [2], [3].

### Case study

A 7-year-old girl from a rural area had been experiencing urinary control issues since infancy characterized by constant overflow incontinence. This was accompanied by gait disturbances. Initially diagnosed by a local general practitioner as scoliosis-related syndromes, she was born to non-consanguineous parents through cesarean delivery at term following an uneventful pregnancy by a non-diabetic mother.

During the examination, the patient was observed in a wheelchair with an abnormal gait. She could perform hip flexion and abduction movements, but the movement of her foot was severely affected. The patient exhibited significant hypoesthesia in the lower limbs and the genital area coupled with anal hypotonia. Complete blood count, renal function tests, and serum electrolytes revealed normal results.

**Fig. 1:**
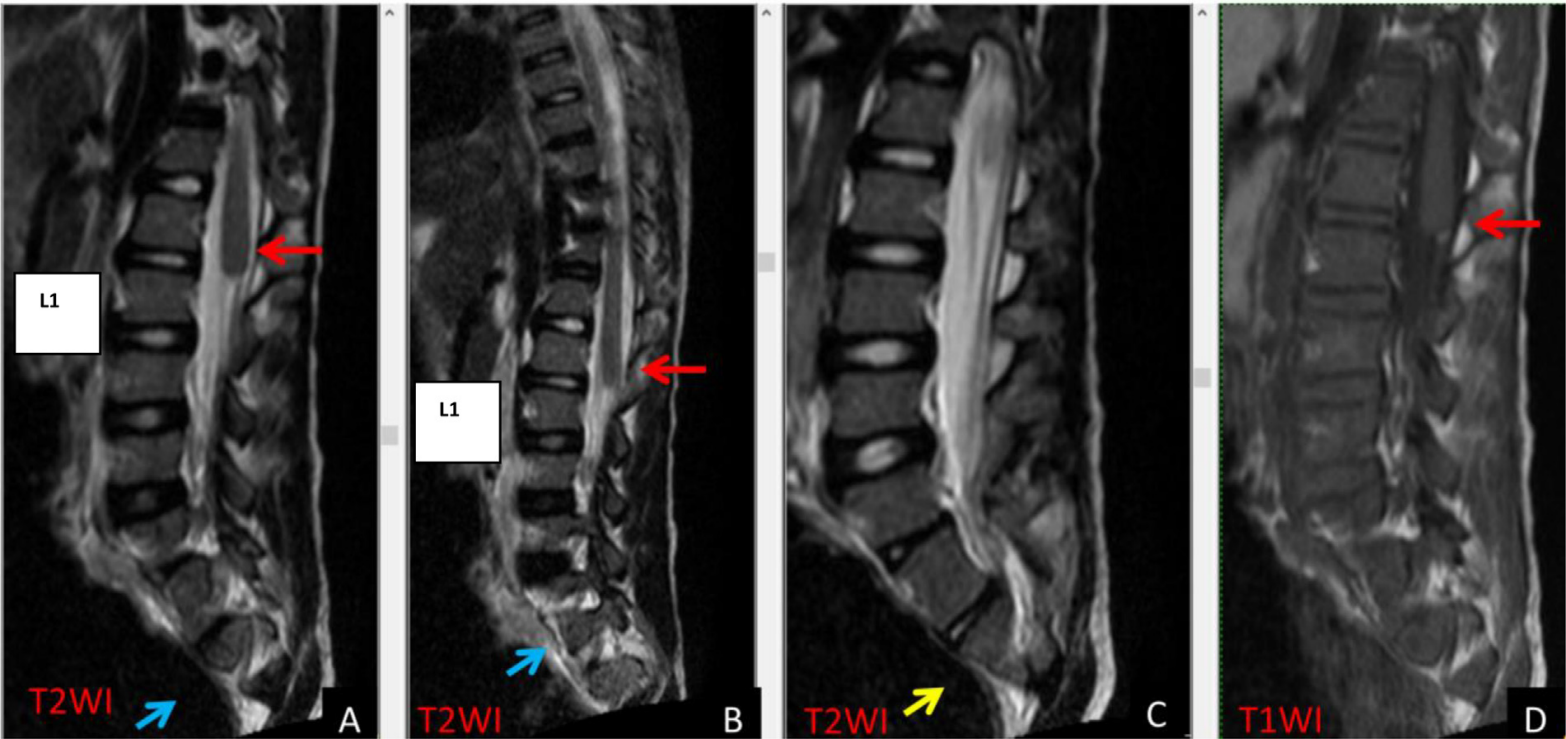
T2WI sagittal (A-C) and T1WI (D) shows a blunt ending of conus medullaris above L1 (A, B, & D). There was partial agenesis at the left side of S1 (blue arrow) versus the right side of S1 (yellow arrow). The findings are consistent with CRS group 1.

**Fig. 2:**
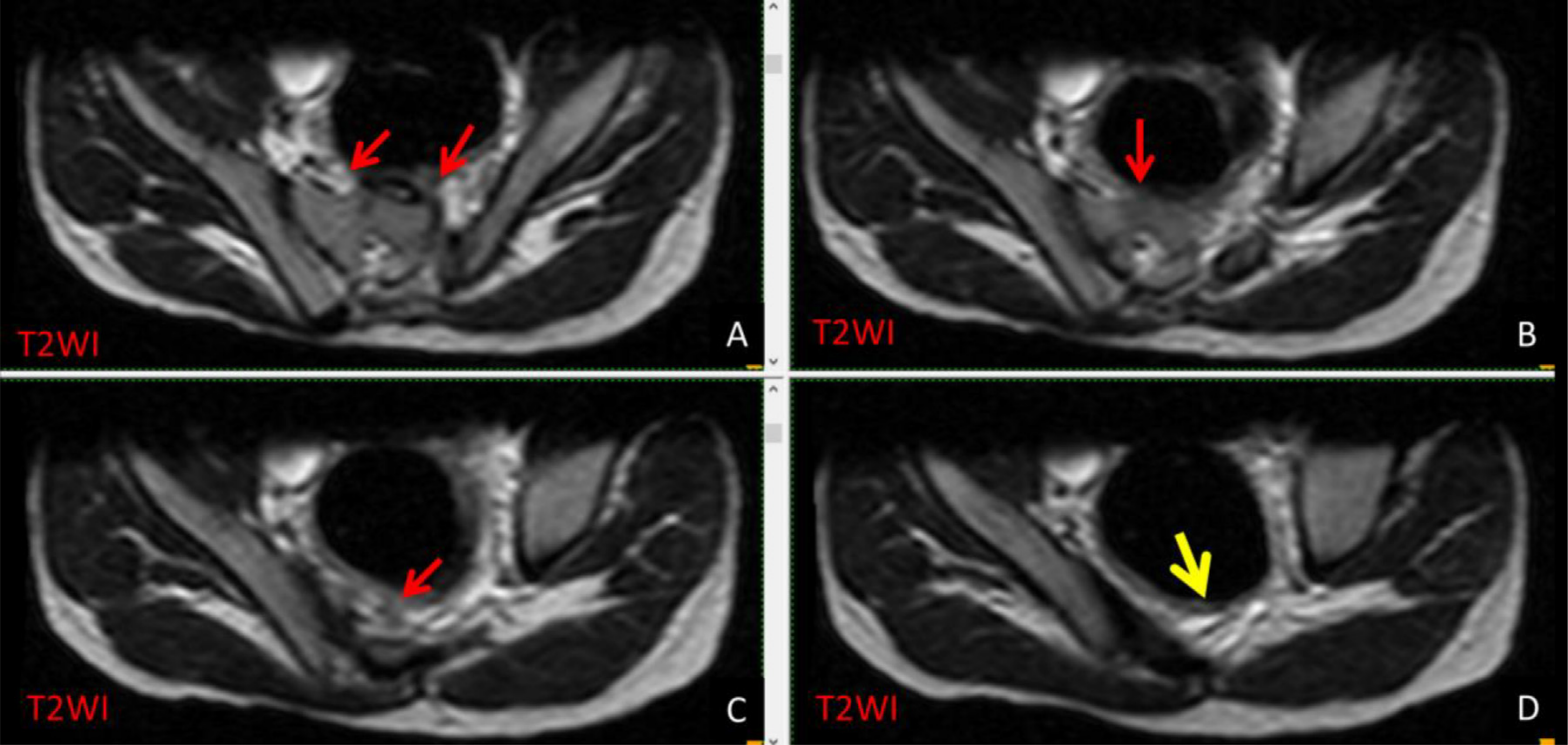
T2WI axial series (A-D) reveals partial agenesis at the left side of S1 (red arrows). There was obvious total agenesis of the rest of the sacral vertebrae (yellow arrow).

**Fig. 3:**
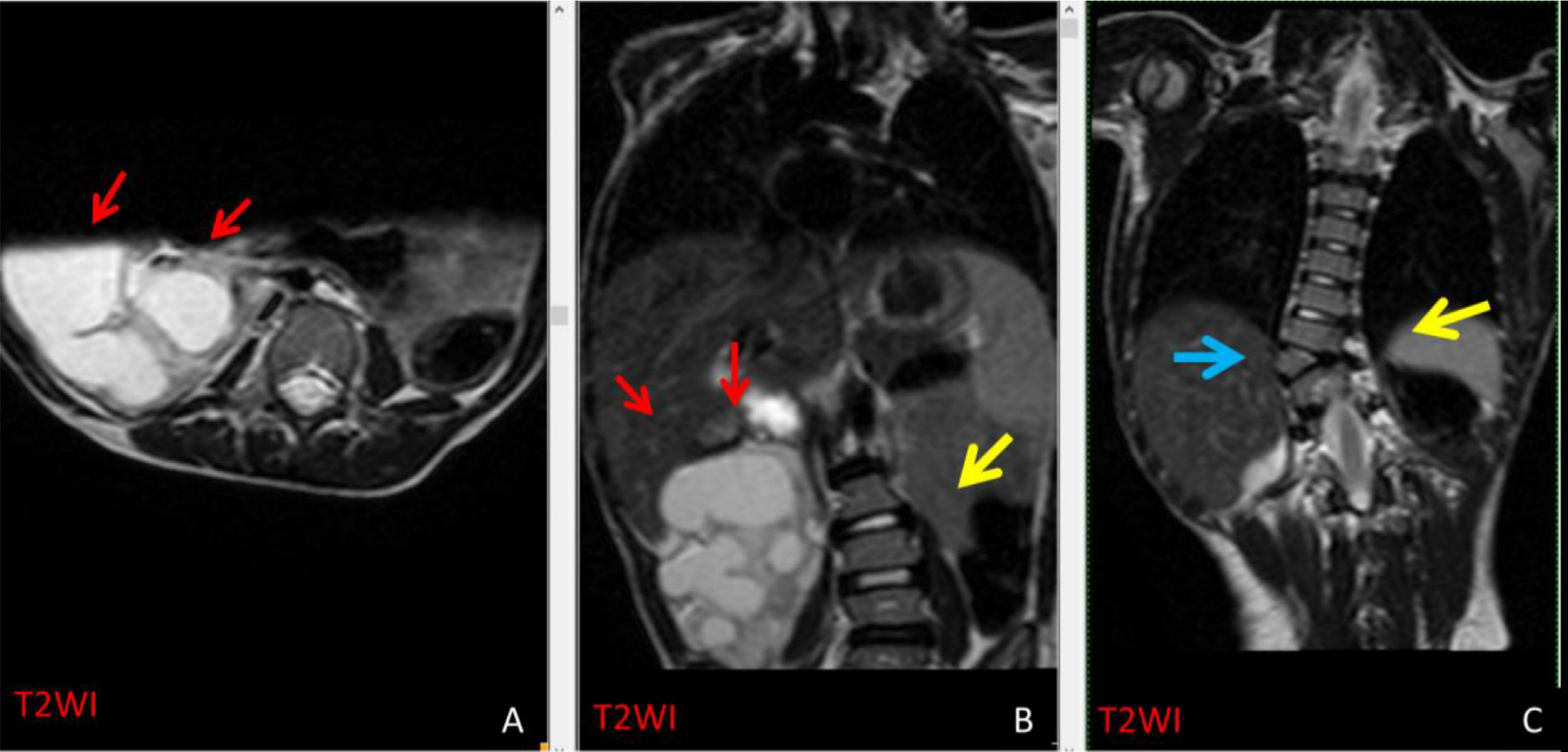
T2WI axial (A) and T2WI coronal (B and C) reveals severe hydronephrosis at the right kidney (red arrows in A&B). In addition to the S-shaped scoliosis at the dorso-lumbar spine (yellow arrow B&C) that is associated with right D9 hemivertebra (blue arrow C).

The MRI scan (Fig. 1, Fig. 2, Fig. 3) showed that the conus medullaris was blunt and terminated above the normal level at D12, while the remainder of the spinal cord was unremarkable. The T2-weighted images show CSF flow artifacts with decreased signal intensities—particularly behind the cord. The cerebellar tonsils extend to the foramen magnum but remain within normal limits.

A hemivertebra was observed at D9 on the right side. This was accompanied by an S-shaped scoliosis in the dorso-lumbar spine. The L5 was sacralized, and there is partial agenesis on the left side of S1 with total agenesis of the remaining sacrum apparent in axial images. The right kidney showed severe hydronephrosis and appeared to be rotated to the right side while the left kidney was not identifiable.

We decided to postpone the surgery for the hemivertebra due to the relatively mild scoliosis presentation. Rather, we referred the child to the urology department to address her kidney deformities.

## Discussion

CRS is a rare congenital condition characterized by arrested caudal spine growth or sacral agenesis [4]. Cases within families suggest a genetic origin with a 5% recurrence risk in families where a child has already been affected. The transmission of CRS may occur through either dominant or recessive characteristics. The dominant form, known as Currarino syndrome, is associated with sacral agenesis and is linked to the HLXB9 gene at 7q36 [5].

Caudal regression may be linked to embryological developmental stages, specifically neurulation 1 and 2. The secondary neural tube forms through the aggregation of cells in the caudal cell mass organizing into a cord-like mass known as the medullary cord. This cord maintains continuity with the primary neural tube, thus forming small cavities that merge into a singular lumen [6]. Maternal diabetes increases the risk of congenital malformations. Studies in mice reveal that exposure to retinoic acid (RA) heightens susceptibility to caudal regression in embryos from diabetic mice. This defect is associated with diabetic pregnancies in humans and involves the Wnt-3a gene, thus suggesting an elevated vulnerability to environmental teratogens during diabetic pregnancies [3].

CRS is often associated with complex abnormalities, and these anomalies may lead to serious complications if left untreated [3,7,8]. Individuals with CRS commonly experience heightened occurrences of renal and genitourinary abnormalities with prevalent neurogenic bladder and renal agenesis [9]. The association of hemivertebra with this condition is exceptionally rare with only a few reported cases in the literature [[10], [11], [12], [13], [14]]. This paper presents the case of a 7-year-old girl initially misdiagnosed with CRS exhibiting partial lower limb deficits, incontinence, and scoliosis due to the presence of hemivertebra at D9. CRS has 3 types:

Type 1 is characterized by the conus terminating cephalic to the lower border of the L1 vertebra, resulting in a higher situated club-shaped conus terminalis and a sacral deficit at or above the S1 vertebra. Type 2, on the other hand, displays the conus ending caudal to the lower border of the L1 vertebra, often with a relatively preserved sacrum and identifiable portions of lower vertebrae. However, cases of spinal cord tethering are more common in Type 2 presentations. Additionally, Type 3 entails a complete absence of the sacrum. Our patient falls under Type 3 classification. The clinical spectrum of CRS varies in severity, thus necessitating multiple surgeries and complex medical care with multidisciplinary team support for optimal treatment and outcomes [15,16]. The treatment approach is complex and must address multiple orthopedic issues such as spinal deformities, spinopelvic instability, and lower limb deformities [17]. In our case, we opted to postpone hemivertebra surgery considering the mild scoliosis, and referred the patient to urology for the management of incontinence and kidney deformity.

## Conclusion

In summary, this study sheds light on the radiological and clinical dimensions of CRS linked to dorsal hemivertebra. CRS is a rare genetic disorder impacting a small fraction of newborns. It is marked by the total or partial absence of lower vertebral structures including the sacral spine. The co-occurrence of hemivertebra with CRS is exceptionally rare. The precise etiology of CRS remains elusive, but evidence suggests a genetic predisposition as well as a potential association with maternal diabetes.

## Declaration

All consents were obtained from the legal representative of the patient and from the Ethics Committee at Damascus University Faculty of Medicine. Data are available upon request from the corresponding author. No funds whatsoever were obtained for any of the authors, and there is no conflict of interest for any of the contributors.

## Patient consent

We have obtained written informed consent for publication from the patient or their legal representatives.
